# Numerical Study on the Progressive Damage Behavior of the Interfacial Debonding between Shape Memory Alloy and Polymer Matrix

**DOI:** 10.3390/ma16010168

**Published:** 2022-12-24

**Authors:** Hao Li, Cong Jiang, Zhaogang Yu, Yun Wan, Yunsheng Ma, Zhaoyang Yang

**Affiliations:** 1 School of Civil Engineering and Architecture, East China Jiaotong University, Nanchang 330013, China; 2 Shandong Chambroad Holding Group Co., Ltd., Binzhou 256500, China

**Keywords:** interface performance, finite element analysis, interfacial debonding, shape memory alloy composites, cohesive zone model

## Abstract

The shape memory alloy reinforced composites have promising application potential for aerospace, automotive and biomedical engineering, while the interfacial bonding performance between shape memory alloy and polymer matrix is crucial to the improvement on the mechanical properties. The interfacial bonding mechanical properties are not uniform on the interface between shape memory alloy and the polymer matrix due to the existence of internal defects. Based on the cohesive zone model, an innovative finite element model is proposed to simulate the progressive damage behavior of the interfacial debonding between shape memory alloy and polymer matrix. The good agreement between the numerical results and the available experimental results indicates the validation of the proposed model. The progressive damage and connection of different positions of the interface between shape memory alloy and polymer matrix result in the final interfacial debonding behavior. Further, the effects of the shape memory alloy length-diameter ratio and embedded depth on the interface performance between shape memory alloy and polymer matrix are investigated.

## 1. Introduction

Shape memory alloy (SMA) has been embedded in polymer composites to improve the vibration control [1,2], damping performance [3,4] and impact resistance properties [5,6] for its unique shape memory effect, high damping capacity and superelastictiy. However, the weak interface caused by the poor compatibility between SMA fiber and the polymer matrix limit the function of SMA excellent performance, making negative influences on the improvement of mechanical properties. An experimental and numerical method was adopted by Lei et al. [7] to investigate the macroscopic mechanical behavior of shape memory alloy hybrid composites (SMAHCs) subjected to quasi-static loading. Gaps between SMA fibers and polymer matrix were observed by scanning electron microscopy (SEM), which indicated the weak interface due to the discrepancy in material compatibility. The presence of internal defects would lead to different interfacial bonding properties on the interface between the SMA and polymer matrix.

Different mechanical [8,9] and chemical [10,11] surface treatments have been conducted on NiTi SMA fibers to improve the interfacial bonding performance. Lin et al. [8] introduced ultrasonic mechanical coating and armouring to synthesize a hydroxyapatite-containing coating on a NiTi SMA. Results show that the bombardment can produce a local amorphous structure of the NiTi SMA near the interface between SMA and coating. Coating oxidation induced by laser annealing improves the coating adhesion and corrosion resistance of the SMA. Huang et al. employed diluted hydrogen fluoride to anodize NiTi SMA [11]. The creation of oxides was observed on a NiTi alloy, and the surface roughness and corrosion resistance of the SMA alloy were enhanced. Laser gas nitriding [12] and laser processing [13] were also utilized to enhance the character of the interface. A continuous structure of TiN was observed on the surface of SMA fiber with the laser gas nitriding treatment, and a thicker oxide layer improving the surface roughness was generated by the laser processing method, which improved the interfacial bonding performance between SMA and polymer matrix. Nanoparticles have been also used to improve the interface performance in recent years [14,15,16,17]. The single fiber pull-out test was used by Yang et al. [14] to study the interfacial bonding strength between SiO_2_ nanoparticle coating SMA filament and epoxy matrix. The ultimate debonding strength was effectively increased due to the enhanced surface roughness. Silane coupling agent KH550 and Al_2_O_3_ nanoparticles were used to treat the SMA surface to modify the interface performance in the research of Wang et al. [17]. The interface performance was significantly enhanced owing to the combination of enhanced surface roughness and increased chemical connection. The interfacial bonding performance between the SMA and polymer matrix is enhanced by mechanical processes, chemical treatments or a combination of both methods. However, because of the differences of acid-alkali corrosion degree, chemical reaction intensity and nanoparticles dispersion, there exists a discrepancy between the adhesion mechanical properties on different locations of the interface between the SMA and the polymer matrix, which is indicated by the interfacial debonding damage morphology observed by scanning electron microscopy (SEM) technology.

Recently, the interface performance between the SMA and polymer matrix has been investigated in many studies via numerical simulation [18,19,20,21,22]. A numerical simulation method was developed by Xu et al. to study the impact progressive damage behavior of shape memory alloy hybrid composites [18]. The surface-based cohesive contact model was used to simulate interfacial debonding behavior between SMA and the polymer matrix. The low-velocity impact behaviors of glass fiber reinforced polymer laminates inserted with SMA wires were studied by Wang et al. using experiments and simulations [19]. The bilinear degradation cohesive model was adopted to simulate the interfacial debonding behavior between SMA and the polymer matrix. The damage patterns and mechanisms of SMA hybrid composite laminates were analysed at different initial impact energies. The structural deformation properties of glass fiber reinforced polymer composite beam actuated by different pre-strained indented SMA wires were experimentally and numerically investigated by Yuan et al. [21]. The interface between SMA wire and the surrounding polymer matrix was assumed to be in perfect adhesive condition in the finite element model. Based on the hypotheses of the perfect adhesive condition or homogeneous bonding performance, the interfacial debonding behavior has been simulated in previous research. In fact, the interfacial bonding performances between SMA and the polymer matrix are not uniform at different locations on account of the existence of internal defects, especially the normal SMA wire. Therefore, to predict the interfacial debonding behavior between SMA and the polymer matrix more accurately, the physical phenomenon of the inequable interfacial bonding properties between SMA and polymer matrix should be taken into account, and more efforts on the interfacial debonding behavior between SMA and polymer matrix are needed to be carried out.

This work focuses on the numerical simulations of the interfacial debonding progressive damage behavior between SMA and polymer matrix in single pull-out test. An innovative finite element model is established taking into account of the inequable interface bonding performances between SMA and polymer matrix. Based on the secondary developing function of the general finite element software ABAQUS 6.14 with the PYTHON language, different interfacial bonding properties are randomly assigned on the interface between SMA and polymer matrix, and the interfacial debonding behavior is simulated using the cohesive zone model. The proposed model is evaluated compared with the available experimental results. Furthermore, the influences of the shape memory alloy dimension and embedded depth on the mechanical responses of SMA/epoxy composites are studied.

## 2. Materials Models

### 2.1. Shape Memory Alloy Model

In this paper, the superelastic mechanical behavior of SMA fiber will be described by the uniaxial model based on an idealized stress–strain relationship within the generalized plasticity framework [7]. The stress–strain behavior is regarded as a linear curve during the process of phase transformation, and the stress–strain relation of SMA fiber can be written as

(1) $$\left\{\sigma \right\}=\left[E\right]\left(\left\{\varepsilon \right\}-\left\{\varepsilon^{\mathrm{pt}}\right\}\right)$$

where *σ* and *ε* stand for the total stress and strain of SMA fiber, *E* is the elastic modulus depended on the martensite fraction, *ε*^pt^ represents the transformation strain produced by the phase transformation during the loading–unloading process.

The transformation strain is expressed as [7]

(2) $$\left\{\Delta \varepsilon^{\mathrm{pt}}\right\}=\Delta \xi_{M}\varepsilon_{\max}^{\mathrm{pt}}\frac{\partial P}{\partial \left\{\Delta \sigma \right\}}$$

*ξ*_M_ stands for the martensite fraction, $\varepsilon_{\max}^{\mathrm{pt}}$ represents the maximum transformation strain. The symbol Δ means the increment. Based on the assumption of an isotropic material behavior, the pressure dependency of phase transformation is simulated by introducing the Drucker-Prager function:

(3) $$P=\sqrt{\sigma :M:\sigma }+\lambda \mathrm{Tr}(\sigma )$$

where Tr is the trace operator and *λ* represents a material parameter characterizing the material response in tension and compression, defined as [7]

(4) $$\lambda =\frac{\sigma_{C}^{\mathrm{AM}}-\sigma_{T}^{\mathrm{AM}}}{\sigma_{C}^{\mathrm{AM}}+\sigma_{T}^{\mathrm{AM}}}$$

where $\sigma_{C}^{\mathrm{AM}}$ and $\sigma_{T}^{\mathrm{AM}}$ is the critical stress of phase transformation starting from austenite to martensite during compression and tension.

Considering the two-phase transformation during loading and unloading process: conversion from austenite to martensite (A→M) and conversion from martensite to austenite (M→A). The evolution of the martensite fraction *ξ*_M_ can be expressed as [7]

(5) $$d\xi_{M}=\left\{\begin{array}{cc} -D^{\mathrm{AM}}(1-\xi_{M})\frac{dP}{P-\sigma_{f}^{\mathrm{AM}}(1+\lambda )} & A\to M\\ D^{\mathrm{MA}}\xi_{M}\frac{dP}{P-\sigma_{f}^{\mathrm{MA}}(1+\lambda )} & M\to A \end{array}\right.$$

where *D* describes the direction of transformation, and can be written as

(6) $$D^{\mathrm{AM}}=\left\{\begin{array}{l} 1\;\;\;\;\;\;\;\;\mathrm{if}\left\{\begin{array}{l} \sigma_{s}^{\mathrm{AM}}(1+\lambda )<P<\sigma_{f}^{\mathrm{AM}}(1+\lambda )\\ dP>0 \end{array}\right.\\ 0\;\;\;\;\;\;\;\;\mathrm{otherwise} \end{array}\right.$$

(7) $$D^{\mathrm{MA}}=\left\{\begin{array}{l} 1\;\;\;\;\;\;\;\;\mathrm{if}\left\{\begin{array}{l} \sigma_{s}^{\mathrm{MA}}(1+\lambda )<P<\sigma_{f}^{\mathrm{MA}}(1+\lambda )\\ dP<0 \end{array}\right.\\ 0\;\;\;\;\;\;\;\;\mathrm{otherwise} \end{array}\right.$$

where $\sigma_{s}^{\mathrm{AM}}$, $\sigma_{f}^{\mathrm{AM}}$, $\sigma_{s}^{\mathrm{MA}}$ and $\sigma_{f}^{\mathrm{MA}}$ stands for the starting and finishing stress of phase transformation, respectively.

### 2.2. Interfacial Debonding Model

**Figure 1:**
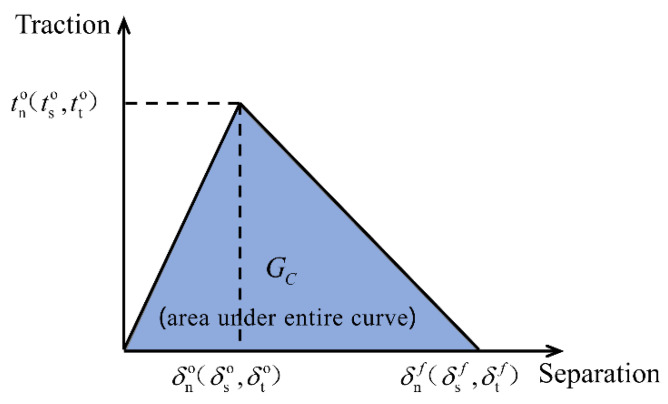
Typical bilinear traction-separation response.

The cohesive zone model (CZM) has been a useful method in simulating adhesives, bonded interfaces, gaskets and rock fracture [23,24,25,26,27,28]. In this paper, the interfacial debonding behavior between SMA and epoxy resin will be predicted by zero thickness cohesive elements randomly generated by the secondary development of the software ABAQUS with the PYTHON language. The bilinear traction-separation response is employed to describe the process of the interfacial debonding initiation and propagation as shown in Figure 1.

The bilinear traction-separation response assumes initially linear elastic behavior followed by the initiation and linear evolution of damage. When the original constitutive thickness of cohesive elements is set to 1.0, the linear elastic behavior can be written as

(8) $$t=\left\{\begin{array}{c} t_{n}\\ t_{s}\\ t_{t} \end{array}\right\}=\left[\begin{array}{ccc} E_{nn} & E_{ns} & E_{nt}\\ E_{ns} & E_{ss} & E_{st}\\ E_{nt} & E_{st} & E_{tt} \end{array}\right]\left\{\begin{array}{c} \delta_{n}\\ \delta_{s}\\ \delta_{t} \end{array}\right\}=E\delta$$

where **t** and **δ** stand for the nominal traction stress vector and separation vector, respectively. The **E** is the interfacial stiffness matrix. The subscripts *n*, *s* and *t* represent the normal and the two shear directions, respectively.

Damage initiation is assumed to occur when a quadratic interaction function relating to the nominal stress ratios reaches the value one. This criterion can be written as

(9) $${\left\{\frac{\langle t_{n}\rangle }{t_{n}^{0}}\right\}}^{2}+{\left\{\frac{t_{s}}{t_{s}^{0}}\right\}}^{2}+{\left\{\frac{t_{t}}{t_{t}^{0}}\right\}}^{2}=1$$

where $t_{n}^{0}$, $t_{s}^{0}$ and $t_{t}^{0}$ stand for the peak values of the nominal stress when the separation is either purely normal to the interface or purely in other two shear directions, respectively. The symbol 〈 〉 is the Macaulay bracket, which signifies that a pure compressive deformation or stress state does not initiate damage.

The damage evolution law depicts the rate at which the interfacial stiffness is degraded once the associated damage initiation criterion is reached. Additionally, the effective stresses defined with the damage variable *D* according to [23] is

(10) $$\begin{array}{l} \bar{t_{n}}=\left\{\begin{array}{c} (1-D)t_{n},t_{n}\geq 0\\ t_{n} \end{array}\right.\\ \bar{t_{s}}=(1-D)t_{s}\\ \bar{t_{t}}=(1-D)t_{t} \end{array}$$

where $t_{n}$, $t_{s}$ and $t_{t}$ calculated based on the (8) expression above, represent the stress components obtained by the linear elastic traction–separation behavior for the current strains without damage.

The damage variable *D* stands for the overall damage in the interface and monotonically increases from zero to one upon further loading after the damage initiation. The value of damage variable *D* larger than zero indicates that the damage of cohesive elements occurs, the value one of damage variable *D* means the final failure of cohesive elements, and the elements will be removed. The damage variable *D* for linear softening based on energy can be defined as [23]

(11) $$D=\frac{\delta_{m}^{f}(\delta_{m}^{\max}-\delta_{m}^{0})}{\delta_{m}^{\max}(\delta_{m}^{f}-\delta_{m}^{0})}$$

where $\delta_{m}^{f}$ is the effective displacement at complete failure relative to $\delta_{m}^{0}$, the effective displacement at damage initiation. $\delta_{m}^{f}=2G^{C}/T_{eff}^{0}$ with $G^{C}$ as the energy dissipated due to failure and $T_{eff}^{0}$ as the effective traction at damage initiation. $\delta_{m}^{\max}$ represents the maximum effective displacement acquired during the loading history.

## 3. Finite Element Model

### 3.1. Pull-Out Test Finite Element Model

**Figure 2:**
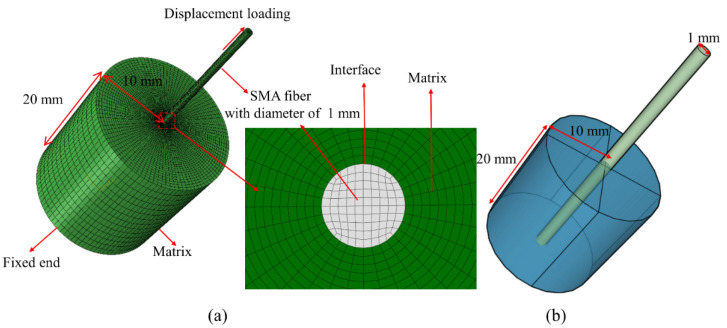
Finite element model for pull-out testing: (**a**) Finite element model and (**b**) actual geometry of the experimental testing.

The damage evolution of interfacial debonding mechanical behavior between SMA and epoxy resin of the single fiber pull-out test was simulated using the commercial finite element software ABAQUS/Explicit. A schematic representation of the finite element model with 20 mm in embedded depth of the SMA fiber is illustrated in Figure 2. Based on the pull-out test, the dimensions of the cylindrical epoxy resin matrix were 20 mm in diameter and 20 mm in height, and the diameter of the SMA fiber was 1 mm. The bottom of the model was fixed in all directions to match the experimental constraint conditions, and a displacement loading of total 0.5 mm at the SMA end in the Z direction was specified to ensure the complete interfacial debonding between SMA and epoxy resin.

The mechanical parameters of the SMA fiber and epoxy resin matrix used in this work are summarized in Table 1 [7,14]. The interfacial bonding properties between SMA and epoxy resin are discussed later in this part.

The SMA fiber and epoxy resin were meshed using the C3D8R reduced integration element, and the zero thickness COH3D8 cohesive elements were established at the interface between SMA and epoxy resin with the ABAQUS-PYTHON scripting language. To achieve high computational efficiency, the element size of models monotonically increased from the central zone at the SMA-epoxy resin interface with 0.1 mm × 0.1 mm elements to the outer edge of the epoxy resin with 0.8 mm × 0.8 mm elements. The general contact was applied to simulate the contact between SMA and epoxy. Once the cohesive elements had completely failed, the dynamic penalty method was employed and the friction contact coefficient was usually set as 0.3 [29,30] for the contact between the metal and polymer composite.

### 3.2. Cohesive Elements with Random Interfacial Properties Generated by PYTHON Language

The approach to create zero thickness cohesive elements with random interfacial performance at the SMA-epoxy interface using the PYTHON scripting language was described in detail as follows: Firstly, the nodal data of each part was read out from the ABAQUS input file, and the part elements were classified and numbered. Based on this information, the sets of Node, Element and Face were created using PYTHON scripting language. Secondly, for each old node, all correlative parts with quantity n in total were identified, and new nodes with quantity (n − 1) in total were thus created. These new nodes were stored in an array New Point for the creation of cohesive elements later. Thirdly, the elements needed to associate at the interface were sought using the getElements function of PYTHON. The coordinates of cohesive elements were acquired utilizing the getNodes function once the interface was obtained, and the nodes of cohesive elements were thus located. The cohesive elements at the interface were thus produced by the Element function of part module in ABAQUS. Fourthly, the new ABAQUS input file including the information of updated Node, Element of parts and cohesive elements was created. Lastly, different interfacial bonding properties were created by the Property module in ABAQUS using PYTHON scripting language; the interface properties produced were randomly assigned to the cohesive elements created by the previous step.

### 3.3. Interfacial Bonding Properties of Cohesive Elements

It is well known that the interfacial debonding behavior between SMA and resin matrix is quite complicated, the local bonding properties on the SMA/matrix interface can be remarkably different for normal SMA fibers due to the existence of internal defects. To simplify the analysis, five kinds of interface properties roughly corresponding to the weak interface performance and to the strong interface performance were properly chosen based on experimental data [14,31] in this work and summarized in Table 2.

**Figure 3:**
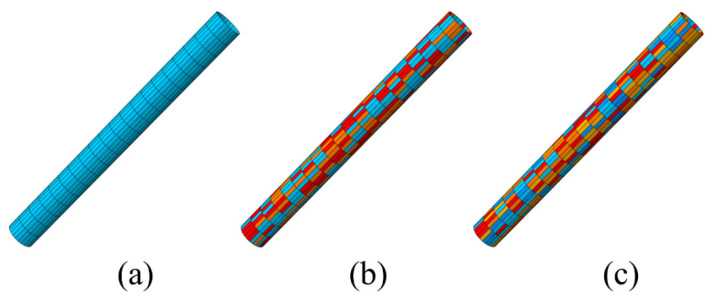
Cohesive elements with the three modes interface performance: (**a**) Mode I with the single medium interface properties, (**b**) Mode II with the combinations of the weaker, medium and stronger interface properties, (**c**) Mode III with all the five kinds of interface properties.

For sufficient analysis and comparison of the influences of different interfacial bonding properties on the SMA/matrix interfacial debonding behavior, three modes of the finite element model with different combinations of the five interfacial bonding properties were established and studied in this work. In detail, Mode I represented the interface cohesive elements assigned by the single medium interface properties, Mode II signified the interface cohesive elements randomly assigned by the weaker, medium and stronger three kinds of interface properties, and Mode III stood for the interface cohesive elements randomly assigned by all the five kinds of interface properties. The zero-thickness cohesive elements with the three different modes interface performance in the finite element model created by PYTHON language were shown in Figure 3.

## 4. Results and Discussion

### 4.1. Simulation Results and Analysis of the Interfacial Debonding Behavior

**Figure 4:**
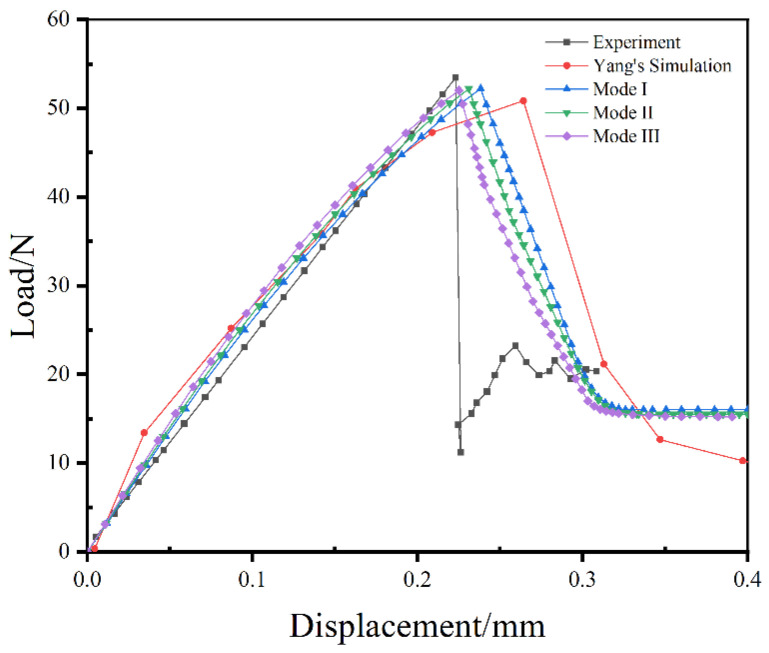
Comparison of the load–displacement curves between experiment testing and finite element simulations.

The load–displacement curves of the SMA single fiber pull-out test from the experiment, Yang’s prediction [14] and the three modes simulations are shown in Figure 4. It can be seen that the trend of the results predicted by simulations reasonably agrees with the experimental results. The load–displacement curve of the experiment mainly include four stages: the initial linear stage; the nonlinear stage until the ultimate load; the stage of the load with a dramatic drop; and the stage of the load approximately reaching a constant value. The load–displacement curves simulated also can be generally divided into four stages: the initial elastic stage; the nonlinear stage before the maximum load; the stage of the load with a gradual decrease process; and the stage of the load attaining to a constant value. As seen, the ultimate load of the experimental test is 53.45 N, and the corresponding displacement is 0.22 mm. In the Yang’s prediction, the ultimate load and the corresponding displacement are 50.83 N and 0.26 mm, respectively, which produces errors of 4.9% and 18.2% for each mechanical response. While for the models proposed in this work, the simulations are in better agreement with the experimental results compared with the Yang’s simulation. The maximum error of the ultimate load is 2.5% for Mode III, and the biggest difference of the displacement associated with the ultimate load is 9.1% for Mode I. Furthermore, based on the mechanical responses and the trend of the load–displacement curves, it is obvious that the Mode III prediction matches best with the experimental results, the Mode II simulation is more consistent with the experimental data compared to the Mode I numerical results. For Mode III, the ultimate load is 52.12 N, only generating a decrease of 2.5% compared to the experimental measurement, and the relative displacement is 0.22 mm, which is consistent with the experimental record. Moreover, the trend of the load–displacement curve is gradually close to the experimental test with more interfacial bonding properties used, especially observed from the stage of the load decrease after the ultimate load.

**Figure 5:**
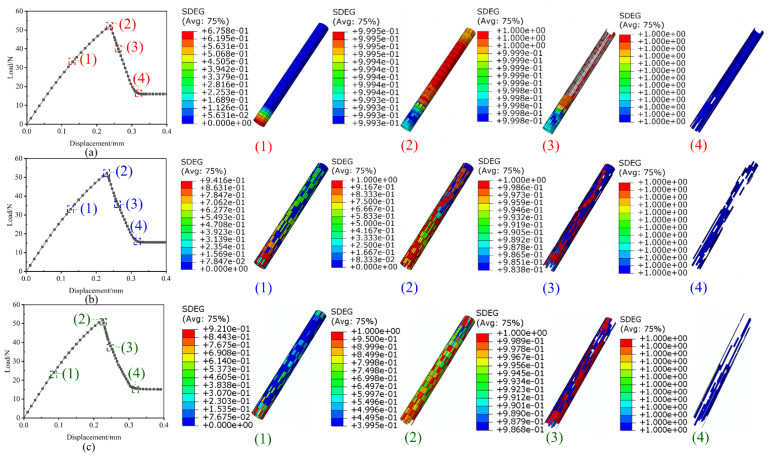
Representative pictures of the damage evolution of the cohesive elements from the three proposed modes: (**a**) Mode I with uniform interfacial bonding properties, (**b**) Mode II with three randomly distributed interfacial bonding properties and (**c**) Mode III with five randomly distributed interfacial bonding properties.

The representative damage initiation and progressive evolution pictures of the zero-thickness cohesive elements created at the interface between SMA and epoxy resin from the three proposed modes are shown in Figure 5. As indicated in the Yang’s work [14], the damage process of the cohesive elements can be divided into four stages, the initial linear stage, the nonlinear deformation stage, the progressive damage stage and the complete failure stage. It is evident that there is no damage appearance in the initial linear elastic stage. The damage initiation of cohesive elements is induced with load increasing in the nonlinear deformation stage. For the Mode I with only single interfacial bonding property, the prediction displays that the damage initiation of cohesive elements is first induced at the bottom of the SMA/epoxy interface because of larger stress caused, which is similar to the simulation results of Yang’s work [14]. For the Mode II and Mode III with multiple interfacial bonding properties, the numerical results show that the damage initiation of cohesive elements occurs at different positions of the SMA/epoxy interface due to the nature of randomly distributed, multiple interfacial bonding properties. After the damage initiation, the progressive damage evolution of cohesive elements occurs at the third stage. The loss of loading bearing capacity of partial cohesive elements results in the decrease of load when completely failed cohesive elements are gradually deleted in the model. For Mode I, the trend of the load shows a nearly linear decrease, which is consistent with the prediction of Yang’s research [14]. The cohesive elements are gradually failed from the bottom to the top of the SMA/epoxy interface due to only single interface performance specified. The SMA/epoxy is finally debonding when all cohesive elements are completely failed. For Mode II and Mode III, the trend of the load presents piecewise linear decrease reasonably. The cohesive elements with weak interface performance first loses carrying capacity, and the failure positions can be randomly distributed due to the nature of the SMA/epoxy interface created in this work. The cohesive elements with strong interface performance then failed with the increase of load. The interfacial debonding behavior between SMA and epoxy appears when different positions of the failed cohesive elements are connected. It is worth noting that there is a discrepancy of the load decrease trend between numerical predictions and experimental test. The interfacial debonding behavior occurs quickly due to the nature of poor compatibility between the normal SMA and epoxy. The load drop can be dramatic as the load bearing capacity is low due to the weak interface performance in experimental test. The friction contact between SMA and epoxy emerges after the occurrence of SMA/epoxy interfacial debonding, and the SMA fiber is pulled out from the epoxy resin gradually.

**Figure 6:**
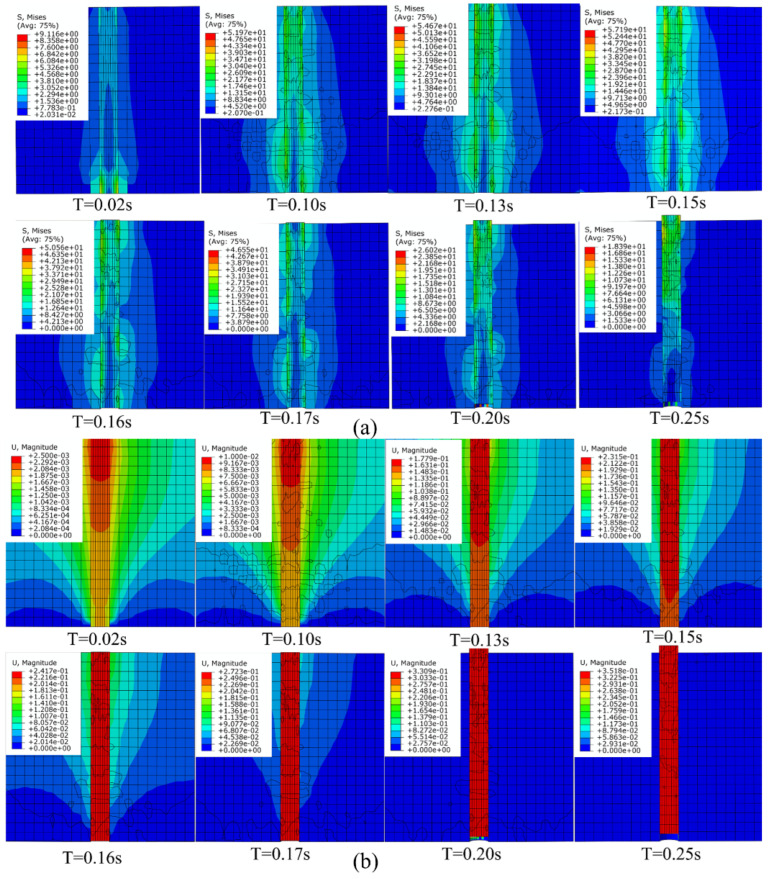
Typical stress distributions versus time (**a**) and deformation versus time (**b**) in the pull-out process of SMA fiber.

The typical stress distributions and deformation of the SMA/epoxy composites versus time in the pull-out process of SMA fiber of Mode III are plotted in Figure 6. It is apparent that the stress distribution and deformation generally present symmetrically around the SMA fiber in the initial linear stage due to the boundary conditions, and the stress is larger at the bottom of the composites relative to other regions such as the representative picture of loading time of 0.02 s. Due to the assignment of different bonding properties on the SMA/epoxy interface, the symmetry of the stress distribution and deformation around the SMA fiber is broken in the nonlinear deformation stage, particularly the occurrence of damage initiation of cohesive elements as the situation of loading time of 0.1 s. Additionally, the phenomenon of stress concentration appears at the locations of weak interfacial bonding properties. With loading increasing, partial-failed cohesive elements are deleted reaching the loading time of 0.15 s; as a result, the drop of the mechanical response of load occurs. The cohesive elements at different positions of the SMA/epoxy interface are gradually failed because of the random distribution of different interfacial bonding properties from the load time of 0.15 s to 0.2 s, and the progressive decrease of the mechanical response load is observed as shown in Figure 4. The interfacial debonding behavior between SMA and epoxy is induced when the failed cohesive elements at different positions are connected like the state of the loading time of 0.2 s. The SMA fiber is gradually pulled out from the epoxy resin matrix after the interfacial debonding damage behavior similar to the condition of the loading time of 0.25 s, and the dominating mechanical response of the SMA/epoxy composite translates to the friction contact behavior until the SMA fiber is completed pulled out.

### 4.2. Simulation of the Composites with Different Embedded Depths

**Figure 7:**
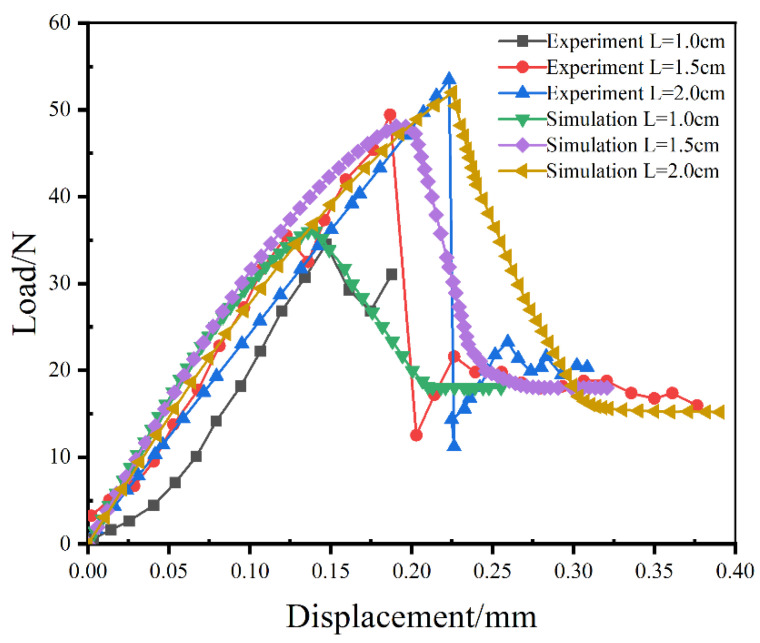
Load versus displacement curves of the experiments and simulations with different embedded depths.

The influences of embedded depth on the mechanical responses of SMA fiber pulled out from the epoxy resin matrix were studied by the Mode III finite element model. The load versus displacement curves of the SMA/epoxy composites with three different embedded depths by experimental testing [14] and numerical simulation are shown in Figure 7. The results from the experiment and simulation all indicate that the mechanical responses of the SMA/epoxy composites with the embedded depths of the 1.0 cm and 1.5 cm are similar with the composite with the 2.0 cm embedded depth described above. The process of SMA fiber pulled out can be divided into four stages for all composites with different embedded depths: the initial linear elastic stage; the nonlinear deformation stage; the progressive interfacial debonding stage; and the friction contact stage. In general, both the ultimate load and the corresponding displacement are increased with the increase of the embedded depth of SMA fiber, and the load shows a very dramatic decrease at the SMA/epoxy interfacial debonding stage and reaches a constant value at the friction contact stage. It can also be seen that the trend of the numerical predictions reasonably agrees with the experimental results. The ultimate load and the corresponding displacement of the SMA/epoxy composite with embedded depth of 1.0 cm from experimental testing are 34.496 N and 0.147 mm, respectively. Compared with the experimental results of composite with 1.0 cm embedded depth, the prediction of the ultimate load shows an increase of 4.27%, and the corresponding displacement has a decrease of 4.76%. For the composite with the embedded depth of 1.5 cm, the ultimate load and the corresponding displacement of experimental record are 49.425 N and 0.187 cm, respectively, and the numerical results of the ultimate load and the corresponding displacement present a decrease of 2.63% and an increase of 1.6%, respectively. Therefore, the reasonably good agreement between the experimental results and the numerical predictions indicates that the proposed SMA/matrix interface bonding finite element model is applicable to this type of simulation.

**Figure 8:**
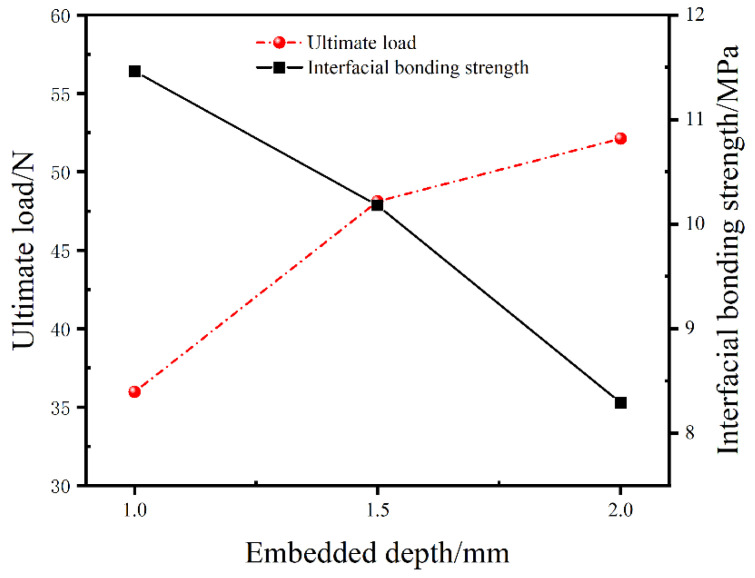
Variation tendencies of the ultimate load and the average interfacial bonding strength versus the embedded depth of the SMA fiber.

The variation tendencies of the ultimate pull-out load and the average interfacial bonding strength of SMA/epoxy composites versus the embedded depth of the SMA fiber are shown in Figure 8. It is evident that the ultimate pull-out load is improved with the increase of the embedded depth, and the average interfacial bonding strength of SMA/epoxy composites is reduced with the embedded depth increasing. In detail, compared to the ultimate load of 35.97 N of the composite with the 1.0 cm embedded depth of SMA fiber, the ultimate load of the composite with the 1.5 cm embedded depth increases by 33.8% and the load with the embedded depth of 2.0 cm shows an increase of 44.9%. For the average interfacial bonding strength of the SMA/epoxy composites, the strengths of the composites with the 1.5 cm embedded depth and with the 2.0 cm embedded depth decrease by 11.2% and 27.6% relative to the composite with the embedded depth of 1.0 cm, respectively. With the embedded depth increasing, the cohesive area between SMA fiber and epoxy resin matrix is increased, thus the overall load bearing capacity of the SMA/epoxy interface is improved. In other words, the ultimate load of the SMA fiber pulled out from the epoxy resin matrix is enhanced. Nevertheless, more internal defects can be induced on the SMA/epoxy interface, and the influence of the weak interface performance between SMA fiber and epoxy resin because of the poor compatibility on the mechanical response of SMA/epoxy composites is more prominent. Hence, the average interfacial bonding strength of the SMA/epoxy composites is decreased.

### 4.3. Analysis of the Composites with Different SMA Dimensions

**Figure 9:**
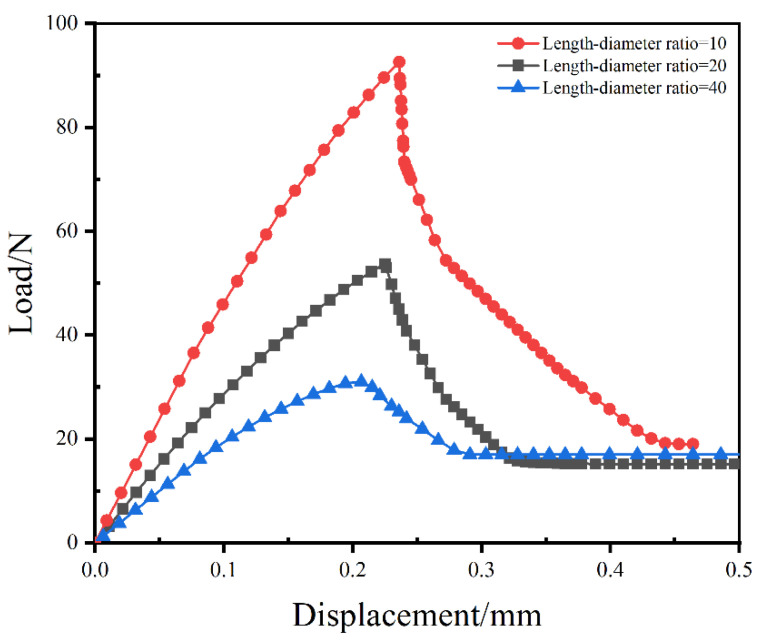
Load versus displacement curves of the SMA/epoxy composites with different length–diameter ratios.

The effects of the length-diameter ratio of SMA fiber on the mechanical responses of SMA/epoxy composites under pull-out loading were analysed using the Mode III model in this section. Three diameters 0.5 mm, 1 mm and 2 mm of the SMA fibers were selected in this paper as the three diameters are commonly used in the pull-out testing of the SMA/epoxy composites. The length of the SMA fiber of the length-diameter ratio just represented the embedded depth. The embedded depth of 2 cm was employed in this analysis, hence three length-diameter ratios: 40, 20 and 10 were produced, corresponding to the diameters 0.5 mm, 1 mm and 2 mm of the SMA fibers, respectively. The load versus displacement curves of the SMA/epoxy composites with the three different length-diameter ratios are plotted in Figure 9. It is evident that the trends of the load–displacement curves are similar to each other independently of the length-diameter ratio of the SMA fiber. Furthermore, the overall trend of the curves is also similar to the composites with different embedded depths. The simulation results indicate that both the ultimate load and the corresponding displacement of the SMA/epoxy composites increase with the length-diameter ratio decreasing. Compared to the increase of the corresponding displacement, the increase of the ultimate load is more apparent. Compared to the composite with the length-diameter ratio of 40, the ultimate load of the composite with the length-diameter ratio of 10 reaches the increase of 198.1%, while the corresponding displacement shows an increase of 18%. The length-diameter ratio of the SMA fiber has a greater influence on the ultimate load than the associated displacement. The final load of each composite reaches a constant value which stands for the friction mechanical response between the SMA and epoxy after the interfacial debonding behavior.

**Figure 10:**
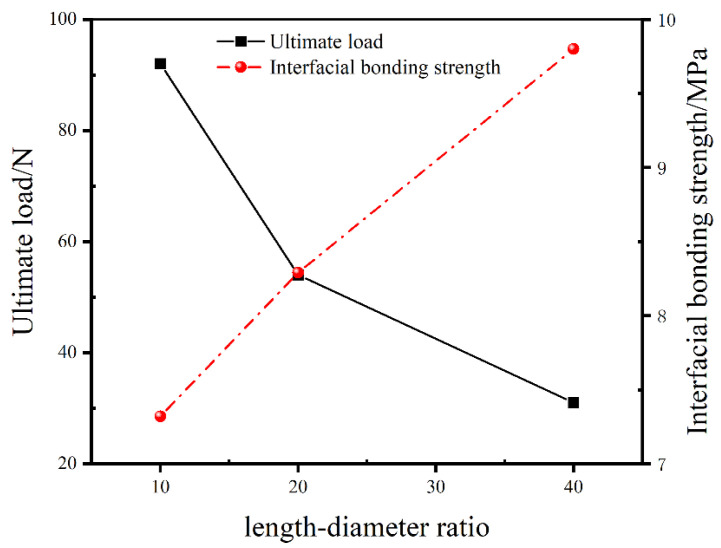
Variation tendencies of the ultimate load and the average interfacial bonding strength versus the length–diameter ratio of the SMA fiber.

The variation tendencies of the ultimate pull-out load and the average interfacial bonding strength of SMA/epoxy composites versus the length–diameter ratio of the SMA fiber are plotted in Figure 10. It is apparent that the ultimate pull-out load is decreased with the increase of the length-diameter ratio, and the average interfacial bonding strength of SMA/epoxy composites is enhanced with the length–diameter ratio increasing. In detail, compared to the ultimate load of 31.05 N of the composite with the 40 length-diameter ratio of SMA fiber, the ultimate load of the composite with the 20 length–diameter ratio increases by 67.9%, and the load with the length–diameter ratio of 10 shows an improvement of 198.1%. For the average interfacial bonding strength of the SMA/epoxy composites, the bonding strengths of the composites with the length–diameter ratios of 20 and 10 decrease by 15.4 % and 25.3% compared to the composite with the 40 length–diameter ratio, respectively. With the length–diameter ratio increasing, the interface bonding area between the SMA fiber and epoxy resin matrix is decreased, hence the overall load bearing capacity of the SMA/epoxy interface is decreased. Namely the load to pull the SMA fiber out from the epoxy resin matrix is decreased, whereas with the diameter of the SMA fiber increasing, or the length-diameter ratio decreasing, internal defects can be more easily induced on the SMA/epoxy interface, the effect of the poor interface performance between SMA fiber and epoxy resin due to the internal defects on the mechanical response of SMA/epoxy composites is more prominent, and the average interfacial bonding strength of the SMA/epoxy composites is decreased. Hence, to acquire SMA/resin matrix composites with high interface performance, more attention should be paid on the improvement of the poor compatibility between SMA fiber and resin matrix.

## 5. Conclusions

This paper aims to investigate the interface performance between SMA and polymer matrix of the shape memory alloy fiber reinforced composites through the finite element method. The interfacial debonding behavior between shape memory alloy and polymer matrix is predicted based on a new cohesive zone model established using the ABAQUS-PYTHON scripting language. The proposed model is validated compared to the available experimental results. From the numerical simulation of the SMA/polymer matrix interfacial debonding behavior, and the analysis of the effects of the embedded depth and dimension of SMA fiber on the mechanical responses of SMA/polymer matrix composites, three main conclusions can be drawn as the following:

1. The interfacial debonding behavior between SMA and polymer matrix is caused by the progressive damage and connection of different positions of the interface with random interfacial bonding properties. A dramatic load drop of the pull-out test is induced by the interfacial debonding behavior between SMA and polymer matrix.
2. The ultimate load and the corresponding displacement of the SMA fiber pulled out from the polymer matrix increase with the increase of the embedded depth, while the average interfacial bonding strength of the SMA/polymer matrix composites is decreased, which is caused by the influence of the weak interface performance due to the poor compatibility between SMA and the polymer matrix.
3. With the length-diameter ratio of the SMA fiber decreasing, the ultimate load and the associated displacement of the SMA/polymer matrix composites is improved, while the average interfacial bonding strength is decreased. Compared to the composites with the length–diameter ratio of 40, the ultimate load and the average interfacial bonding strength of the composites with length–diameter ratio of 10 increased by 198.1% and decreased by 25.3%, respectively.

## Figures and Tables

**Table 1:** The mechanical properties of the SMA fiber and epoxy resin [7,14].

Materials	Density (kg·m^−3^)	Modulus (GPa)	Poisson’s Ratio	$\sigma_{s}^{\mathrm{AM}}\;(\mathrm{MPa})$	$\sigma_{f}^{\mathrm{AM}}\;(\mathrm{MPa})$	$\sigma_{s}^{\mathrm{MA}}\;(\mathrm{MPa})$	$\sigma_{f}^{\mathrm{MA}}\;(\mathrm{MPa})$
SMA	6.45	30	0.32	365.89	412.68	98.83	61.51
Epoxy resin	1.6	3.9	0.39				

**Table 2:** Interface properties between SMA and epoxy.

Parameters	Interface Stiffness (Mpa/mm)			Interface Strength (Mpa)			Interface Fracture Toughness (N/mm)		
	*K_n_*	*K_s_*	*K_t_*	$t_{n}^{0}$	$t_{s}^{0}$	$t_{t}^{0}$	$G_{n}^{c}$	$G_{s}^{c}$	$G_{t}^{c}$
Weaker interface	500	310	310	20	10	10	0.42	0.42	0.42
Weak interface	500	310	310	25	15	15	0.42	0.42	0.42
Medium interface	500	310	310	35	20	20	0.42	0.42	0.42
Strong interface	500	310	310	45	25	25	0.42	0.42	0.42
Stronger interface	500	310	310	50	30	30	0.42	0.42	0.42

## Data Availability

Not applicable.
